# Mutation in Mouse *Hei10,* an E3 Ubiquitin Ligase, Disrupts Meiotic Crossing Over

**DOI:** 10.1371/journal.pgen.0030139

**Published:** 2007-08-31

**Authors:** Jeremy O Ward, Laura G Reinholdt, William W Motley, Lisa M Niswander, Dekker C Deacon, Laurie B Griffin, Kristofor K Langlais, Vickie L Backus, Kerry J Schimenti, Marilyn J O'Brien, John J Eppig, John C Schimenti

**Affiliations:** 1 Middlebury College, Middlebury, Vermont, United States of America; 2 The Jackson Laboratory, Bar Harbor, Maine, United States of America; 3 Cornell University, Ithaca, New York, United States of America; Harvard Medical School, United States of America

## Abstract

Crossing over during meiotic prophase I is required for sexual reproduction in mice and contributes to genome-wide genetic diversity. Here we report on the characterization of an *N*-ethyl-*N*-nitrosourea-induced, recessive allele called *mei4,* which causes sterility in both sexes owing to meiotic defects. In mutant spermatocytes, chromosomes fail to congress properly at the metaphase plate, leading to arrest and apoptosis before the first meiotic division. Mutant oocytes have a similar chromosomal phenotype but in vitro can undergo meiotic divisions and fertilization before arresting. During late meiotic prophase in *mei4* mutant males, absence of cyclin dependent kinase 2 and mismatch repair protein association from chromosome cores is correlated with the premature separation of bivalents at diplonema owing to lack of chiasmata. We have identified the causative mutation, a transversion in the 5′ splice donor site of exon 1 in the mouse ortholog of Human Enhancer of Invasion 10 (*Hei10;* also known as Gm288 in mouse and CCNB1IP1 in human), a putative B-type cyclin E3 ubiquitin ligase. Importantly, orthologs of *Hei10* are found exclusively in deuterostomes and not in more ancestral protostomes such as yeast, worms, or flies. The cloning and characterization of the *mei4* allele of *Hei10* demonstrates a novel link between cell cycle regulation and mismatch repair during prophase I.

## Author Summary

Human infertility and reproductive complications have devastating social and monetary costs. Errors in meiosis during reproduction may lead to birth defects, spontaneous abortion, or infertility. Many of the genes essential for meiosis function in DNA repair and mutations in several of these genes have been shown to contribute to cancer. The identification of the genes necessary for normal meiosis is an important goal and will potentially influence the fields of reproductive and cancer biology. In this study, genetic screens in mice have generated the mutation *mei4*. *mei4* causes male and female sterility by disrupting meiosis and altering the function of the DNA repair system known as mismatch repair. We have identified the causative mutation behind the mei4 phenotype in a gene called Human Enhancer of Invasion 10 or *Hei10*. This work demonstrates that *Hei10* is essential for the completion of meiosis and that it functions to coordinate the DNA repair system and the progression of the cell cycle during meiosis.

## Introduction

The successful completion of meiosis I (MI) in the vast majority of multicellular eukaryotes requires replication of the genome, proper signals from somatic support cells, synapsis, and crossing over between homologous chromosomes. The progression of the distinct phases of MI is thought to be orchestrated by periodic synthesis and degradation of cyclins, the activity of cyclin dependent kinases (CDKs), the correct establishment of the synaptonemal complex (SC), and the proper functioning of the DNA repair machinery during replication and recombination. The accuracy of the meiotic cell cycle in mammals is monitored both during recombination via the pachytene checkpoint in prophase I or during the spindle checkpoint at metaphase I (reviewed in [1,2]). While meiocytes of both sexes are sensitive to early recombinational repair defects, the pachytene checkpoint in males is more sensitive to synapsis defects per se than in females [2]. The components of the mammalian pachytene checkpoint and the nature of the fundamental molecular communications triggering cell cycle arrest after recognition of DNA anomalies in prophase I remain elusive.

The DNA repair machinery is essential for meiotic progression. Genetically programmed double strand breaks (DSBs) are generated and processed by SPO11 [3,4]. DSB formation is followed by recombinational repair facilitated by the RecA homologs RAD51 and DMC1, whose protein products colocalize with ∼300 early recombination nodules on chromosome cores during leptonema [5,6]. Both are subsequently replaced by the single-stranded binding protein replication protein A (RPA) [5,6]. Foci containing the MutS homolog MSH4 colocalize with RAD51 foci during zygonema and persist into the onset of early pachynema [7,8]. Roughly half of MSH4 foci appear to be involved in reciprocal recombinations between homologous chromosomes, while the others result in non-crossover gene conversion events [9]. MSH4 and MSH5 form a heterodimeric complex thought to mediate recognition of the Holliday junction [10]. Both the *Msh4* ^−*/*−^ and *Msh5* ^−*/*−^ knockout mice fail to synapse appropriately and arrest in pachynema [7]. Temporally, Msh4 and Msh5 mark the first appearance of the mismatch repair (MMR) system in prophase I.

During pachynema, the protein products of the MutL homologs *Mlh3* and *Mlh1* [6,11,12] colocalize with the sites of reciprocal recombination and mark the second appearance of the MMR system in prophase I. MLH3/MLH1 foci appear in early pachynema and are present until early diplonema. Targeted deletion of *Mlh3* causes arrest at metaphase I and prevents MLH1 recruitment to the chromosome [11]. Meiocytes deficient in MLH1 fail to complete meiosis owing to loss of bivalent maintenance and disorganized univalents at metaphase I [13–16]. As evidenced by their respective knockout phenotypes, the spatiotemporal progression from foci containing MSH4/MSH5 to those containing MLH3/MLH1 appears to be a critical event in the transition from susceptibility to a pachytene checkpoint to that of a spindle checkpoint. Interestingly, little is known about the cell cycle-related molecular mechanisms that are responsible for this transition.

In addition to the role of the DNA repair machinery, meiosis is regulated in part by the synthesis and ubiquitin-mediated degradation of cyclins and their functional relationship with CDKs. There are three cyclins (A1, B1, and B3) that are necessary for or are specifically expressed during mammalian meiosis (reviewed in [17,18]). Several CDKs have been implicated in the progression of meiosis, including CDK1, CDK2, CDK4, and CDK6 [19]. CDK2 and CDK4 are essential for proper meiosis as both *Cdk2* ^−*/*−^ and *Cdk4* ^−*/*−^ mice are sterile [20–22]. *Cdk2* ^−*/*−^ spermatocytes arrest in mid-pachynema, whereas *Cdk2* ^−*/*−^ oocytes are lost perinatally [20]. *Cdk4* ^−*/*−^ mice have reduced viability and display degeneration of primary spermatocytes [21]. Interestingly, CDK2 colocalizes with MLH1 at sites of reciprocal recombination [23]. Beyond this, the role of CDK2 during the progression of MMR in prophase I remains unclear.

Ubiquitin-mediated proteolysis is thought to ensure the properly timed involvement of the cyclins in the meiotic cell cycle. For example, limited proteolytic degradation of meiotic cyclin B1 (CCNB1) is necessary for homolog disjunction and release from MI and the exit from MII in yeast and mammals [17,24]. Ubiquitin-mediated proteolysis involves transfer of ubiquitin by a ubiquitin-activating enzyme (E1) to a ubiquitin conjugating enzyme (E2), which in turn assembles a ubiquitin chain onto a substrate recruited by a substrate-specific E3 ligase (reviewed in [25]). To date however, very few interactions have been identified between substrate-specific E3 ligases and meiotic cell cycle regulators. One such potential interaction involves the anaphase-promoting complex and CCNB1. The anaphase promoting complex is a large multiprotein complex containing E3-ubiquitin ligase activity thought to be responsible for degradation of meiotic cyclins, including CCNB1 [26].

Evidence is emerging that ubiquitin conjugation by non-anaphase promoting complex-related pathways occurs during gametogenesis. Male sterility results from targeted disruptions of the ubiquitin-like DNA repair gene *Rad23b* or the *Ubr2* ubiquitin ligase via failure to initiate spermatogenesis and failure to complete MI, respectively [27,28]. Further, mutations in *Hr6b* (mouse *RAD6* homolog) [29], *Siah1a* [30], and F-box protein family members result in MI defects [31], particularly in males. Despite these phenotypes, none of the above mentioned genes have been directly connected phenotypically or mechanistically to the progression of the cell cycle machinery during meiosis.

Previously, we reported the results of forward genetic screens to identify mutant alleles of genes necessary for the early stages of MI that lacked recognizable orthologs in other model organisms [32]. Here we report on the characterization of one of these alleles called *mei4*. Mutant spermatocytes are defective in maintaining interhomolog associations at MI due to a lack of crossing over. Positional cloning led to the identification of a mutation in the mouse ortholog of Human Enhancer of Invasion 10 *(Hei10)*. This gene encodes a putative E3 ligase that has not previously been implicated in meiotic recombination. Defects in *Hei10* result in aberrant MMR protein and CDK localization. We proposed that the function of *Hei10* during MI reveals a link between cell cycle regulation and the accurate resolution of reciprocal recombination intermediates.

## Results

### Meiosis Is Abnormal in *mei4/mei4* Males and Females

**Figure 1:**
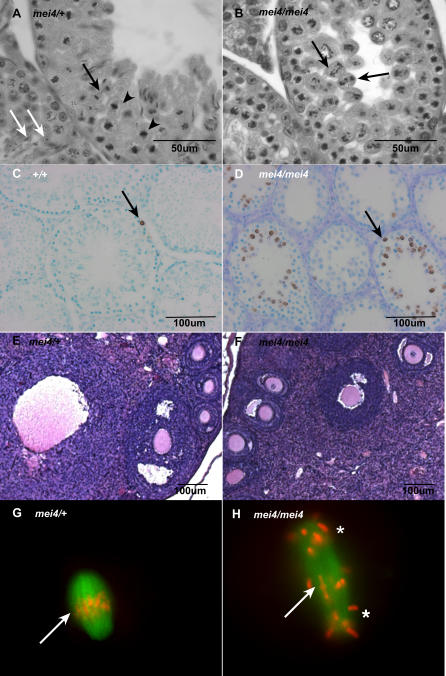
The *mei4* Mutation Inhibits Normal Meiotic Chromosome Alignment at Metaphase I in Males and Females and Is Associated with Apoptosis in Spermatocytes (A) Heterozygous (*mei4/+)* seminiferous tubule cross sections stained with hematoxylin and eosin show normal telophase and metaphase I structures (black arrow and arrowheads, respectively) as well as postmeiotic round spermatids and elongating differentiating spermatids (white arrows). (B) Homozygous mutant (*mei4/mei4*) sections show abnormal metaphase and anaphase I-like spermatocytes with disrupted chromosome congression to the metaphase plate and aberrant chromosome distribution on the spindle (black arrows). (C and D) *mei4/mei4* mutant seminiferous tubules have significantly greater numbers of apoptotic cells (brown cell, black arrow) than *+/+* tubules (D versus C, respectively) as determined by TUNEL assay. The average number of TUNEL positive cells per 0.146-mm^2^ field in tubules that contained apoptotic cells was 140 for *mei4/mei4* versus 9.6 for +/+ (*p* = 3.28 × 10^−15^). (E and F) The ovaries of *mei4/mei4* females appear histologically normal. Hematoxylin and eosin-stained cross sections reveal follicles and oocytes in all stages of development in normal (E) and mutant animals (F). (G and H) Hoechst-stained metaphase I chromosome structures occur normally in *mei4/+* oocytes (white arrow, G) and abnormally (asterisks, H) in *mei4/mei4* oocytes. Infrequently, normal metaphase chromosomes structures are seen in mutant oocytes (white arrow, [H]). Spindles in (G and H) are labeled with an anti-b tubulin antibody.

*mei4/mei4* male and female mice are sterile and do not exhibit any other obvious somatic defects. Mutant males produce no sperm, have small testes (unpublished data), and spermatocytes undergo a metaphase I arrest (Figure 1A and 1B) [32]. In contrast to normal mice (Figure 1A), mutant testes (Figure 1B) contained no round, elongating (Figure 1A, white arrows), or further differentiated spermatids. Unlike the normal telophase and metaphase structures present in *mei4/+* controls (Figure 1A, black arrow and black arrowheads, respectively), mutant metaphase spermatocytes contained abnormal metaphase and anaphase-like cells (Figure 1B, black arrows). The incidence of apoptosis was significantly higher in *mei4/mei4* versus wild-type (*+/+*) tubules (black arrows, Figure 1D versus 1C) (*p* = 3.28 × 10^−15^). The gross morphology and histopathology of ovaries from *mei4*/+ and *mei4/mei4* females did not differ (Figure 1E and 1F). Heterozygous and mutant oocytes matured in vitro resumed meiosis as characterized by metaphase plate formation at MI (Figure 1G, white arrow). However, like spermatocytes, most chromosomes in *mei4/mei4* oocytes failed to congress to, and align at, the metaphase plate (Figure 1H, white asterisks). Bivalent alignment was rare but did occur (Figure 1H, white arrow).

Despite severe metaphase abnormalities, *mei4*/*mei4* oocytes developed into two-celled embryos at a frequency similar to *mei4/+* oocytes (63% versus 80.2% respectively; *p* = 0.1769). However, only 5.6% of *mei4/mei4* two-cell embryos developed into blastocysts, versus 74% of the two-cell stage embryos from *mei4/+* oocytes (*p* = 0.0001) (Table 1). Therefore, the developmental competency of embryos derived from *mei4*/*mei4* oocytes was lost between the two-cell and blastocyst stage.

**Table 1:**
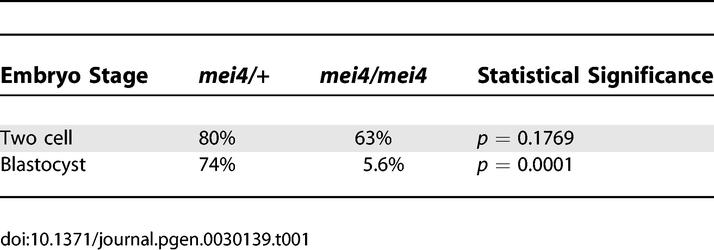
Summary of In Vitro Fertilization

### Defective Interhomolog Associations in Mutant Spermatocytes

**Figure 2:**
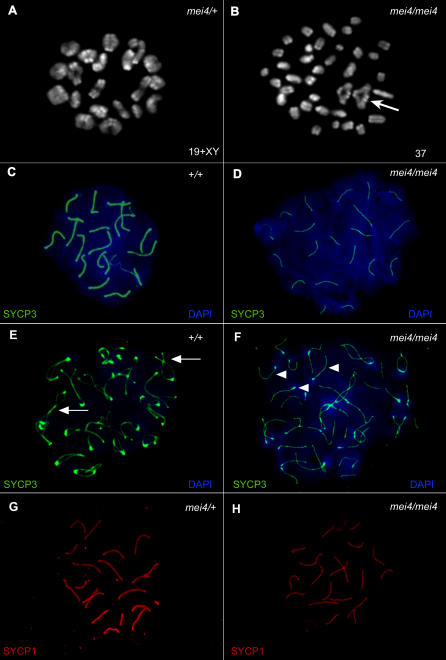
*mei4/mei4* Spermatocytes Synapse in Prophase I but Have Few Intact Bivalents at Metaphase I (A and B) Metaphase chromosome spreads were prepared from normal (*mei4*/+ [A]) and mutant (*mei4*/*mei4* [B]) males and stained with DAPI. In (A) *mei4*/+ spreads exhibit the expected number of bivalent recombination structures. (B) depicts a *mei4/mei4* spread with few chiasmata (white arrow) and with most chromosomes present as univalents. (C–F) Chromosome spreads from normal (+/+) and mutant (*mei4/mei4*) spermatocytes were labeled with antibodies to SYCP3 and counter-stained with DAPI at various stages of MI. Pachytene (C and D) +/+ and *mei4/mei4* spermatocytes are similar. At diplonema, +/+ (E) spreads show pairs of homologous chromosomes associated at sites of recombination (white arrows) and or centromeres. In contrast, in *mei4/mei4* spreads (F) many chromosomes are univalents associated neither at the centromere nor sites of recombination (white arrowheads). The loss of bivalent cohesion is not complete however, and some chiasmata are seen in *mei4/mei4* preparations. (G and H) Chromosome spreads from heterozygote (*mei4/+* [G]) and homozygous recessive (*mei4/mei4* [H]) animals were labeled (red) with antibodies to SYCP1, a marker of the central element and synapsis. In both cases SYCP1 is present indicating that the transverse elements of the SC are forming and that chromosomes in *mei4/mei4* spermatocytes synapse normally in pachynema.

To determine the cause of the aberrant MI in *mei4*/*mei4* spermatocytes, we prepared metaphase spreads. Whereas nineteen metaphase bivalents were present in *mei4/+* spreads (Figure 2A), *mei4/mei4* spreads contained primarily univalents (Figure 2B). Bivalents were rarely present (Figure 2B, white arrow). The total number of condensed chromosomes (univalents + bivalents) approached 40. These observations suggest that either homologous chromosomes never paired and/or synapsed or did so but failed to remain attached upon entry to metaphase.

To examine synapsis, we used antibodies to detect the distribution of SC proteins along mutant and wild-type prophase I chromosomes. SYCP3 marks the axial elements of the SC [33]. Wild-type (*+/+*) and *mei4*/*mei4* spermatocyte chromosome spreads labeled with anti-SYCP3 antibodies revealed the presence of pachytene stage nuclei containing 19 paired chromosomes plus an apparent X-Y pair (Figure 2C and 2D, respectively). However, there was premature homolog separation in diplotene *mei4*/*mei4* spermatocytes (Figure 2E) versus +/+ (Figure 2F). The distribution of SYCP1, a SC transverse element protein that is indicative of synapsis [6,34], was normal in *mei4*/*mei4* versus *mei4/+* spermatocytes (Figure 2H versus 2G).

### Recombination Initiates Normally, but *mei4* Spermatocytes Fail to Form Chiasmata

**Figure 3:**
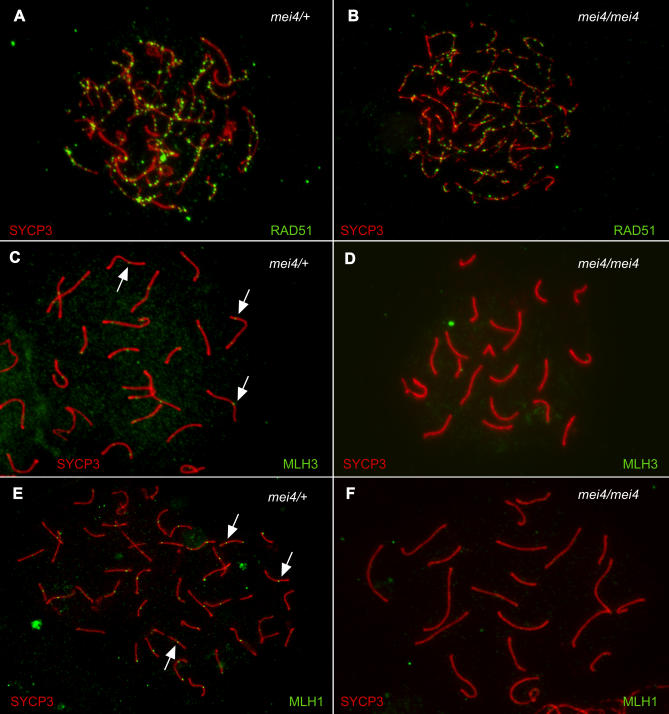
Chromosomes in *mei4/mei4* Spermatocytes Contain RAD51 Foci in Zygonema but Lack MLH3 and MLH1 Foci in Pachynema (A and B) The DSB repair protein RAD51 appears in foci (green) on zygotene chromosomes in both *mei4/+* and *mei4/mei4* chromosome spreads counter labeled with anti-SYCP3 (red). (C–F) Chromosome spreads from heterozygous (*mei4/+*) and mutant (*mei4/mei4*) spermatocytes were labeled with antibodies to MLH3 (green [C and D]) or MLH1 (green [E and F]) and counter-labeled with anti-SYCP3 (red). MLH3 and MLH1 foci (white arrows) are present on *mei4/+* chromosomes (C) and (E) but not on *mei4/mei4* chromosomes (D) and (F).

Since mutant spermatocytes are capable of homolog synapsis but defective in maintaining interhomolog attachment, we examined the progress of recombination in these nuclei. RAD51 foci were distributed similarly along zygotene chromosome cores in control and *mei4*/*mei4* spermatocyte nuclei (Figure 3A and 3B), indicating that DSBs formed and that recombinational repair was initiated. We then examined the distribution of two markers of recombination nodules, the MMR molecules MLH3 and MLH1 [6,11]. Mid-pachytene *mei4/+* spermatocytes had 1–2 MLH3 foci on each synapsed chromosome (Figure 3C) and ∼23 per nucleus, typical for wild-type meioses [6]. However, no MLH3 foci were evident in *mei4/mei4* (Figure 3B). Similar results were obtained with MLH1 (Figure 3E and 3F).

### Candidate Gene Identification and Positional Cloning of *mei4*

**Figure 4:**
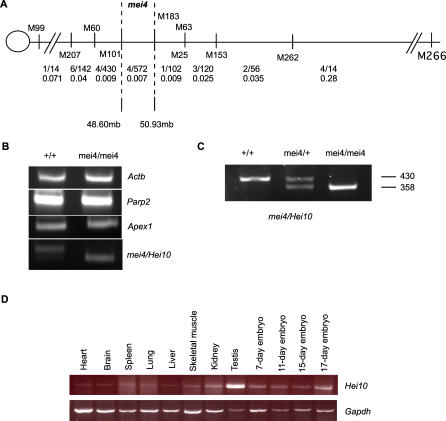
*mei4* Maps to a 2.5-mb Region on Chromosome 14 and Is Associated with an Altered *Hei10* Transcript That Is Expressed Prominently in the Testis and 17-d Embryo (A) The diagram shows microsatellite loci abbreviated with “M” for D14Mit. To map *mei4* genetically, intersubspecific intercrosses were set up between animals heterozygous for *mei4*. The number of meioses scored for recombinants in particular intervals are indicated in raw numbers (# of recombinants/# of meioses) and recombination fractions. The data placed *mei4* in a 0.7-cM region corresponding to a physical distance of ∼2.5 mb. (B) RT-PCR was performed on total RNA isolated from normal (+/+) and mutant (*mei4/mei4*) animals. Neither *Parp2* nor *Apex1* showed altered transcript amount or size (rows 2 and 3, respectively). The *Hei10* transcript is expressed at similar levels in +/+ and *mei4/mei4* but displays altered mobility in agarose gel electrophoresis (row 4). Similar b-Actin (*Actb*) expression in both +/+ and *mei4/mei4* indicates equal RNA amounts (row 1). (C) RT-PCR of +/+, *mei4/+*, and *mei4/mei4* animals. The +/+ (lane 1) displays the expected 430-bp band, whereas the *mei4/+* (lane 2) displays both the expected size band and a smaller band (358 bp) that is identical in size to the only band in the *mei4/mei4* sample (lane 3). (D) A mouse cDNA panel was used to determine the expression pattern of *Hei10* across a range of tissues. Expression relative to the GAPDH control was highest in testis and 17-d-old embryo. The 17-d-old embryo is a mixed male and female sample and represents the onset to midterm of prophase I in the mouse oocyte.

*mei4* was previously mapped to a large region on Chromosome 14 between the markers *D14Mit99* and *D14Mit266* (Figure 4) [32]. In this study, we refined the genetic region containing *mei4* to a 0.7-cM (four recombinants out of 572 meioses) region corresponding to 2.33 Mb between the markers *D14Mit101* and *D14Mit183* on Chromosome 14 (Figure 4A). The mouse Ensembl database (http://www.ensembl.org, version 43, NCBIM36) annotates 62 Refseq genes (Table S1) and 11 other Ensembl annotated genes in this region including a cluster of 28 olfactory receptors. Potential candidate genes for *mei4* were prioritized on the basis of known or predicted meiotic function and/or germ tissue expression pattern. For novel genes or genes of unknown function, protein-protein BLAST (blastp) comparisons were made to identify functional domains or regions of homology to known genes or motifs. Three genes warranted further attention under these criteria: poly ADP ribosylase 2 *(Parp2),* apurinic/apyrimidic endonuclease 1 *(Apex1),* and the Ensembl novel gene Gm288 (Table S1, numbers 33, 35, and 41 in bold-face type). We performed reverse-transcriptase (RT)-PCR on testis RNA from *mei4/+* and *mei4/mei4* animals to detect possible expression variations in *Parp2* and *Apex1* (Figure 4B, rows 2 and 3, respectively). Primer pairs spanning multiple exons were used for both genes. Both genes appeared to be expressed normally in control and mutant testes. We sequenced all exons of both genes and no mutations were found.

### The *mei4* Allele of *Hei10* Contains a Splice Site Mutation

Gm288 encodes the mouse ortholog of human *CCNBP1IP1* (CCNB1 interacting protein 1), also called *HEI10* [35]. Using RT-PCR, we found an altered length transcript present in both *mei4/+* and *mei4/mei4* mutants versus +/+ animals (Figure 4B, row 4). Using primers specific to exons 1 and 2, the expected 430-bp product was seen in +/+ and *mei4/+* animals. In addition, a 358-bp fragment was also present in *mei4/+* heterozygotes (Figure 4C). In *mei4/mei4* testes, we observed only the 358-bp product (Figure 4C). RT-PCR analysis of multiple tissue types revealed that *Hei10* is transcribed prominently in the testes and 17-d embryo (corresponding to prophase I in females) and to a much lesser degree in other tissues (Figure 4D).

**Figure 5:**
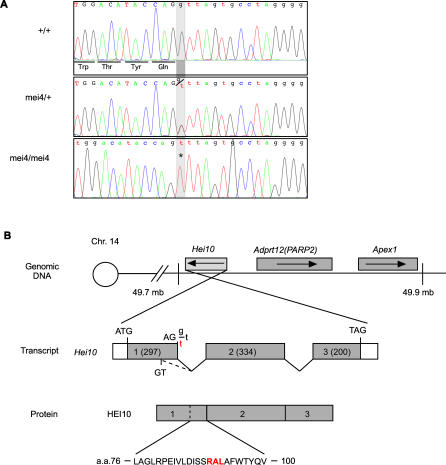
Genomic Sequencing of *Hei10* Reveals a Mutation in the Consensus GT 5′ Splice Site in Intron 1 (A) Genomic DNA from *+/+, mei4/+*, and *mei4/mei4* animals was sequenced, and a single base transversion (g > t, gray shading) was found in the 5′ splice site consensus GT sequence. Exon sequence is denoted with capital letters and intron sequence with lower case. (B) The 5′ splice site of intron 1 contains the consensus GT dinucleotide (*Hei10*). The splice site mutation described in (A) (g > t, red t indicates mutant form) causes the splicing machinery to select a GT site 72 bp upstream of the original site (dashed line). The 72-bp in-frame section of exon 1 is removed with intron 1 and is not present in the mature transcript (sequence not shown). The predicted protein sequence of HEI10 in *mei4/mei4* animals contains a deletion of amino acids (a.a.) 76–100 including a conserved putative cyclin binding domain (red RAL).

Sequencing of *Hei10* genomic DNA from control and mutant mice revealed a G>T transversion at bp 298 (from the start of the first coding exon, G298T) in *mei4/mei4* animals (Figure 5A), which corresponds to the 5′ splice donor site at the 3′ end of exon 1 (Figure 5B, Transcript). Sequencing of cDNA from control and mutant animals showed that G298T causes the use of a cryptic splice donor site 72 bp upstream (GT in position 226) in exon 1. This results in an in-frame, 24 amino acid deletion in the predicted protein product (Figure 5B, Protein, amino acids 76–100) that removes a putative cyclin/CDK interaction domain, the RXL motif (Figure 5B, red RAL) [35–39]. We will refer to the above-described allele as *Hei10^mei4^*.

### Abnormal Localization of CDK2 in *mei4/mei4* Spermatocytes

**Figure 6:**
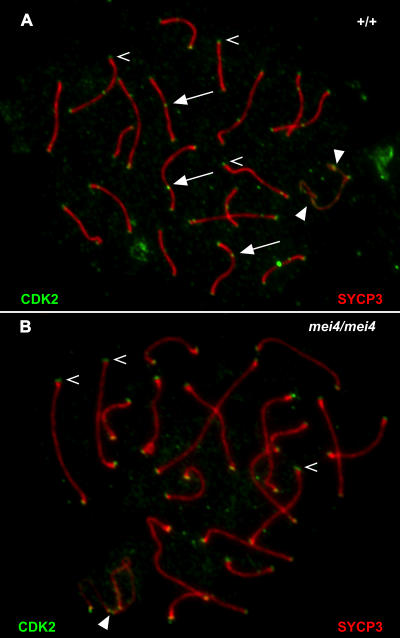
CDK2 Does Not Localize to Interstitial Sites on Synapsed *mei4/mei4* Bivalents during Pachynema (A) Wild-type (+/+) spermatocyte chromosome spread labeled with antibodies against CDK2 (green) and SYCP3 (red). CDK2 localizes to telomeric foci (open arrowheads), along the asynapsed axes of the sex chromosomes (closed arrowheads), and at interstitial sites (white arrows) along synapsed wild-type bivalents in pachynema. (B) *mei4/mei4* spermatocyte chromosome spread labeled as in (A) showing absence of CDK2 localization at interstitial loci. Despite loss at interstitial sites, CDK2 localization is observed at telomeric foci (open arrowheads) and at loci along the sex chromosomes (closed arrowheads).

To investigate the possibility of an error in the progression of the cell cycle, we examined the localization of CDK2, a CDK essential for meiosis [20], on spermatocyte chromosomes (Figure 6). CDK foci are normally observed at the telomeres, at one to two interstitial sites on each synapsed bivalent, and on the asynapsed portions of the X and Y chromosomes in males [23]. Further, CDK2 colocalizes on pachytene chromosomes with MLH1 [23]. Mid-pachytene +/+ spermatocytes displayed CDK2 foci at interstitial sites (Figure 6A, white arrows), telomeres (Figure 6A, open arrowheads), and the X-Y body (Figure 6A, closed arrowheads). In contrast, CDK2 foci were seen at telomeric sites and on the X-Y body on *mei4*/*mei4* pachytene chromosomes (Figure 6B, open arrowheads and white arrow, respectively) but were rarely seen at interstitial sites.

## Discussion

### Failure to Form Chiasmata Is Correlated with Defects in the MMR System

In this study, we have demonstrated that *mei4/mei4* spermatocytes undergo a uniform early metaphase I arrest marked by aberrant chromosome congression (Figure 1). Mutant oocytes show a similar configuration but fail to arrest at early metaphase I and are competent, postfertilization, to reach the two-cell embryonic stage. Immunocytochemical labeling of microspread spermatocytes and 4′ , 6-diamidino-2-phenylindole, dihydrochloride (DAPI) labeling of metaphase chromosomes demonstrates that maintenance of bivalents is lost in all but a few chromosome pairs. Our data show that the high prevalence of univalents in late diplonema and metaphase I are due to a lack of chiasmata.

Failure to form chiasmata can arise from defects in the DSB system, the MMR system, or in the complexes required for proper SC formation [4,11,15]. Normal localization of RAD51 and SYCP3/SYCP1 in *mei4/mei4* spermatocyte nuclei indicates that DSB repair and SC formation are not disrupted. However, MLH3 and MLH1 fail to form foci in *mei4/mei4* spermatocyte nuclei, suggesting that defects in the MMR system cause chiasmata failure and subsequent metaphase arrest in males (Figure 3).

### Loss of the HEI10 RXL Domain May Lead to Inability to Associate with Cyclins

**Figure 7:**
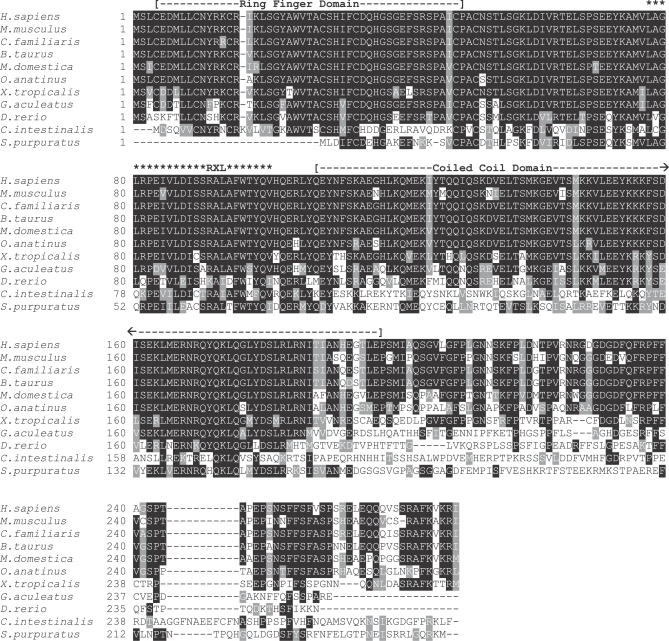
ClustalW Protein Sequence Alignment of HEI10 Orthologs from Deuterostome Lineages Sequence from Homo sapiens (human) and Mus musculus (mouse) have been experimentally determined, while the sequences from Canis familiaris (dog), Bos taurus (cow), Monodelphis domestica (opossum), Ornithorhynchus anatinus (platypus), Xenopus tropicalis (clawed frog), Gasterosteus aculeatus (stickleback fish), Danio rerio (zebrafish), Ciona intestinalis (transparent sea squirt), and Strongylocentrotus purpuratus (California purple sea urchin) were available in databases as predicted proteins (accession numbers are listed below). A candidate RING domain, a potential RXL-type B-type cyclin interaction motif (RAL), the deleted portion of the *Hei10^mei4^* allele (asterisks), and a central coiled-coil domain are indicated with text above the sequences. Using database and BLAST searches, HEI10 orthologs could not be identified in the chicken, yeast, or in any protostomes such as D. melanogaster or C. elegans.

Sequencing of *Hei10^mei4^* cDNA identified a shortened transcript produced in the testes. When translated, this mRNA would produce a protein missing 24 amino acids (residues 76–100) in a highly conserved portion of the N-terminal region of HEI10 (Figure 7). This missing region includes a RXL motif (asterisks, amino acids 91–93, RAL) with predicted cyclin/CDK-binding activity [35]. RXL domains are found in CDK substrates such as p107, p21^CIP1^, and the retinoblastoma protein [36,40–44]. Deletion or mutation of the RXL domain or peptides containing the RXL motif inhibit phosphorylation of the CDK substrate and/or eliminate binding. On the basis of data from a cyclin A/CDK2 crystal structure, the RXL domain contacts a highly conserved remote (relative to the active site) binding domain on the cyclin partner [45]. While we cannot rule out the contributions of the other residues in the region, deletion of the RXL domain in *Hei10^mei4^* would abrogate binding of the E3 to its presumed target, a B-type cyclin.

### *Hei10^mei4^* and the Cyclins

Alternatively, HEI10 may function as a cell cycle regulator that acts between early and late pachynema. The loss of MLH3/MLH1 foci may be secondary to a possible cell cycle error such as premature exit from G2 before chiasmata are established. As mentioned, cyclins A1, B1, and B3 are essential for meiosis. Deletion of the germ-cell specific cyclin A1 results in a MI spermatogenic arrest in males [46,47]. Cyclin A1 mRNA and protein are expressed primarily from late pachynema through diplonema of prophase I [46,47]. CCNB1 is expressed from mid-pachynema through postmeiotic spermiogenesis [48], and regulation of partial proteolytic degradation of this cyclin is necessary for homolog disjunction in mouse oocytes [24]. In oocytes, CCNB1 is essential for the successful completion of MI as loss of *Ccnb1* expression causes accelerated polar body extrusion and inability to enter G2–metaphase meiosis II [49]. Recently, a third mammalian B-type cyclin, cyclin B3 *(Ccnb3)* has been identified that is highly expressed in prepachytene spermatocytes and the fetal ovary [18].

HEI10 has been shown to associate with exogenously expressed human CCNB1 in mitotic cells and in yeast two-hybrid assays utilizing a somatic cell-derived library [35]. Golemis and colleagues suggested that the human protein product of HEI10 functions as a mitotic E3 ubiquitin ligase for CCNB1, ensuring its degradation and thus transition from metaphase I to interphase of meiosis II [35]. In the male germline, however, CCNB1′s activity is highest in postmeiotic spermatids [48], which is temporally inconsistent with a potential role of HEI10 as a CCNB1 E3 ligase. Further, since *Ccnb1* and *Hei10* are expressed in many somatic cell types (*Hei10*, Figure 4D) and mutation of *Ccnb1* causes embryonic lethality [50], one might expect a more pleiotropic somatic phenotype in the *Hei10^mei4^* mutant mice if CCNB1 were the primary target.

Koff and colleagues identified mammalian CCNB3, a meiotic B-type cyclin that is expressed maximally during the leptotene to zygotene transition [18], a point just prior to the first observed phenotypic defect in *Hei10^mei4^* homozygotes. This raises the possibility that CCNB3 is the target of HEI10′s B-type cyclin E3 ligase activity in meiosis. *Hei10^mei4^* has a deletion in the putative cyclin-B interaction domain that contains an RXL motif. We speculate that this mutation abrogates an association between HEI10 and CCNB3. This scenario may explain the observed defects in crossing over, homolog attachment, and congression to the metaphase plate. Alternatively, normal HEI10 may target any of a number of cyclin-B related sequences [51] found in the genome but as yet unstudied in meiosis.

In females, where anaphase I begins despite these errors, missegregation is the inevitable result. Interestingly, the *Hei10^mei4^* phenotype in oocytes is similar, but not identical, to that of *Mlh3* ^−*/*−^ and *Mlh1* ^−*/*−^ mice. Only 7% of *Mlh3* ^−*/*−^ and 13.6% of *Mlh1* ^−*/*−^ oocytes reach the two-cell stage, and none reach the four-cell stage [11,15], whereas 63% of *Hei10^mei4^* zygotes reach the two-cell stage with a small portion (5.6%) even capable of forming a blastocyst. This difference may suggest that HEI10 is not directly involved in MMR but rather serves to couple MMR to the cell cycle machinery. Further, in *Mlh3* ^−*/*−^ and *Mlh1* ^−*/*−^ mice, expression of both proteins is abrogated. Currently, we have no data suggesting that MLH3 or MLH1 protein levels are altered in *Hei10^mei4^* mice, only that their localization with respect to the chromosome cores has changed. Noncore-associated MLH3 and MLH1 may be present and may affect the progression of late prophase or metaphase I in *Hei10^mei4^* females.

### Roles of HEI10 in Meiosis versus Mitosis

The genetic and cytological data presented here are supportive of a key role for *Hei10^mei4^* in mouse meiosis. Toby and colleagues previously demonstrated a role for (human) HEI10 in the mitotic cell cycle [35]. Our data show that *Hei10* is transcribed in many somatic tissues but at considerably lower levels than in testes (Figure 4D). While the effect of the mutation in meiosis is dramatic, we have not identified any obvious defects in *Hei10^mei4^* mice other than infertility. One possible explanation is that while *Hei10* is expressed in many tissues at lower levels than in testis, its hypothesized in vivo target (CCNB3) is primarily expressed in germ cells. Thus, the effects in somatic cells may be subtle.

Alternatively, as a putative B-type E3 ligase, HEI10 may play an essential role maintaining accurate euploid segregation at the meiotic prophase I-metaphase I boundary and the mitotic G2–metaphase boundary. Errors in either, even if relatively rare, could lead to aneuploid segregation and/or neoplasia. Notably, Mine and colleagues have shown that *HEI10* resides at a fusion breakpoint in some uterine leiomyomas, a benign tumor of the reproductive system [52]. We have not noticed an elevated tumor incidence in *Hei10^mei4^* mice.

### *Hei10* Is Conserved among Deuterostomes

Several years ago, we initiated a forward genetic screen with the goal of identifying novel genes required for gametogenesis, with an emphasis on those required for meiosis [32]. Although meiosis is a highly conserved process, it is noteworthy that two (*mei4* and *Mei1* [53]) mutations cloned from these screens do not have direct orthologs in *Saccharomyces cerevisiae, Drosophila melanogaster,* or Caenorhabditis elegans. *Hei10* is conserved among deuterostomes but has not been found in protostomes or unicellular organisms and may serve as a marker of the modification of sexual reproduction during a major transition in the animal lineage. Among deuterostomes, sequence identity is highest in the N-terminal portion of the protein containing the RING finger and a coiled coil domain (Figure 7, amino acids 1–197). The C-terminal portion (residues 198 to the end) of the protein exhibits reduced sequence identity when compared to the N terminus. Interestingly, *Hei10* orthologs have not been found in Gallus gallus or Taeniopygia guttata (zebra finch). These results may reflect incomplete sequence coverage in the *Hei10* syntenic region in chicken or zebra finch or may be the result of divergent meiotic evolution in avian taxa. The identification of *Hei10^mei4^* and *Mei1* validate the efficacy of *N*-ethyl-*N*-nitrosourea screens in mammals as a tool to identify novel genes required for meiosis.

### CDK2 and Prophase I

The *Hei10^mei4^* phenotype sheds light on the role of CDK2 in meiotic progression. *Cdk2* ^−*/*−^ mice fail to complete MI because of an arrest in early prophase I [20] by presumably triggering the pachytene checkpoint. CDK2 foci are normally found at telomeric sites from zygonema through diplonema but at interstitial crossover sites and the unsynapsed portion of the X and Y chromosomes only from mid-pachynema through early-diplonema [23]. In *mei4/mei4* males, which arrest at metaphase I, CDK2 is normally localized to the telomeres and sex chromosomes but, interestingly, is absent at interstitial sites (Figure 6B). Therefore, in the *mei4/mei4* background, the presence of CDK2 at the telomeres and/or the XY body, but not at the interstitial sites, is necessary to bypass the pachytene checkpoint. The correlation of the loss of CDK2 and MLH3/MLH1 foci at interstitial sites on *mei4/mei4* spermatocyte chromosome cores suggests a role for CDK2 mediation of MMR during prophase I.

### CDK2 and Recombination Nodules

**Figure 8:**
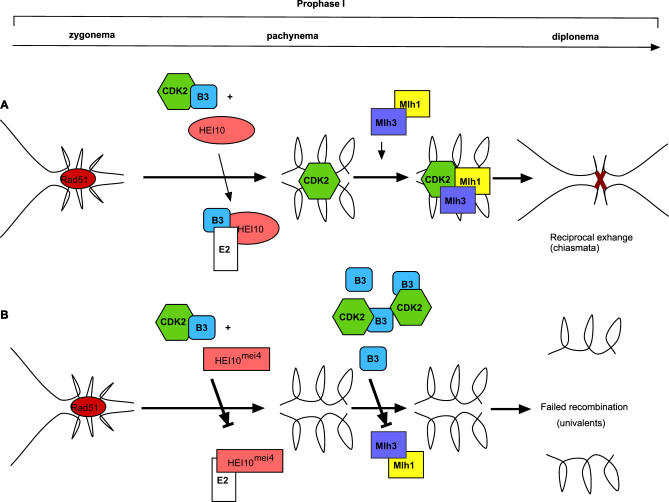
Proposed Mechanism: HEI10^mei4^ Mediated Failure to Degrade CCNB3 Leads to Inability to Recruit MLH3 and MLH1 Resulting in Failed Recombination (A) During normal Prophase I, subsequent to DSB repair via RAD51, HEI10 mediates the degradation of CCNB3 (B3) freeing CDK2 to associate with interstitial sites on chromosome cores in pachynema. Subsequently (or contemporaneously), MLH3 and MLH1 are recruited to recombination nodules containing CDK2. In diplonema, MMR has occurred, MLH3, MLH1, and CDK dissociate from the cores and chiasmata maintain homolog association until the onset of anaphase I. (B) In *mei4/mei4* animals, DSB formation and RAD51 foci occur normally. Inability of HEI10^mei4^ to associate with B3 leads to the accumulation or mislocalization of B3 and the titration of the available CDK2. CDK2 is unable to associate with sites of recombination, as are MLH3 and MLH1. In our model, failure to correct mismatches during Holiday junction resolution leads to incomplete recombination intermediate resolution and arrest at the metaphase I spindle checkpoint.

*Hei10^mei4^* provides evidence of a functional connection between the cell cycle machinery via CDK2 (and possibly via B-type cyclins) and the DNA repair machinery during prophase I. The *Hei10^mei4^* variant has a deletion in the proposed B-type cyclin interaction domain (RXL). CCNB3 has been shown to interact with CDK2 [18]. CDK2 also interacts with MLH1 at sites of recombination in mid-late pachynema [23] and is essential for meiosis [20]. We envision a mechanism by which HEI10 mediates the destruction of CCNB3, permitting CDK2 association with MLH1 or other MMR proteins in recombination nodules on the chromosome cores (Figure 8A). As suggested by Ashley and colleagues, this interpretation is consistent with CDK2 having a substrate in the recombination nodules [23]. In our model, *HEI10^mei4^* fails to recruit CCNB3 to the E2 ubiquitin conjugating enzyme causing the accumulation or mislocalization of CCNB3 during early pachynema. Elevated levels of CCNB3 may preclude CDK2 association with the interstitial sites on chromosome cores and association with the MMR machinery leading to a failure of the MMR system during recombination (Figure 8B). We are currently testing these hypothesized interactions in vitro. The precise in vivo order of these events and molecular interactions involving CCNB3 are not well resolved and await the development of high-quality antibodies to CCNB3.

In conclusion, we have demonstrated a novel mouse mutation (*Hei10^mei4^*) that disrupts chiasmata formation and contributes to aberrant meiotic chromosome congression in mice via an interruption of both cell cycle machinery and the MMR system. We have linked the defects to *Hei10,* a putative E3 ubiquitin ligase with B-type cyclin specificity. They also suggest a role for CDK2 as regulator of recombination competence. These results provide new insights into the mechanistic control and the cell cycle regulation of mammalian meiotic DNA repair systems.

## Materials and Methods

### Mice.

Mice were obtained from and maintained at The Jackson Laboratory and the Middlebury College Research Animal Facility according to the procedures outlined by the IACUC committees at both institutions.

### Oocyte culture and assessment of meiotic progression.

Ovaries were removed from females 44 h after treatment of 3-wk-old female mice with equine chorionic gonadotropin (Organon, http://www.organon.com) and manually disrupted to release oocytes. The released oocytes were cultured as described to test for spontaneous resumption of meiosis [54,55]. The oocytes were examined using a stereomicroscope after 15 h of culture and assessed for germinal vesicle breakdown, indicative of the resumption of meiosis and the presence of a polar body, which is usually characteristic of progression to metaphase II.

### Fertilization and blastocyst culture.

Oocytes at metaphase II were washed three times in minimum essential medium (MEM) supplemented with 3 mg/ml BSA. In vitro fertilization and culture were performed as described previously [56,57]. Eggs were removed from fertilization drops after 4–6 h, rinsed twice in 2.5 ml MEM, and cultured overnight in a borosilicate tube with 1 ml of fresh medium. At 25 h postfertilization, cleavage-stage embryos were rinsed twice with KSOM medium supplemented with essential and nonessential amino acids and 1 mg/ml BSA (KSOM/AA) and cultured to the blastocyst stage in 1 ml KSOM/AA medium in borosilicate tubes.

### Histology.

Testes were fixed in Bouin's solution for >24 h before being embedded in paraffin. We cut 5-mm sections, and they were stained with hematoxylin and eosin (H & E). Digital images were obtained with either an Olympus BX51 upright microscope fitted with an Olympus MagniFire CCD (The Jackson Laboratory) or a Zeiss Axioskop 2 *plus* microscope fitted with a Zeiss Axiocam MRm digital camera using AxioVision 4.4 software (Middlebury College Imaging Facility).

### Immunocytochemistry.

Testes from prepubertal males 17–24 d postpartum (dpp) were microspread and immunolabeled as described previously [58,59] and counterstained with DAPI (Molecular Probes, http://probes.invitrogen.com) to visualize DNA. Antibodies used were anti-b tubulin (Sigma, http://www.sigmaaldrich.com), anti-SYCP3 (0.8mg/ml) (Abcam, http://www.abcam.com), anti-MLH3 (1:500) (gift from P. E. Cohen), anti-MLH1 (1:50) (BD Biosciences, http://www.bdbiosciences.com), anti-CDK2 (1:300) (Santa Cruz Biotechnology, http://www.scbt.com), anti-SYCP1 (1:500) (Abcam), and anti-RAD51 (1:600) (Calbiochem, http://www.emdbiosciences.com).

### Immunofluorescent detection of the oocyte meiotic spindle assembly.

Oocytes were collected, cultured (described above), fixed, and stained for DNA and b-tubulin as previously described [60].

### Detection of apoptosis.

Testes were obtained from *+/+*, *mei4*/+, and *mei4/mei4* mutant mice that were between 2 and 4 mo old. Apoptosis in paraffin embedded testes was assessed using ApopTag Plus Peroxidase In Situ Apoptosis Detection kit (Chemicon, http://www.chemicon.com). Tissue section images were taken using an Axiovert 200 microscope and AxioCamMRc camera using AxioVision 4.4 software. Apoptotic cells were imaged and counted in 0.146-mm^2^ fields of view (200× total magnification) that were representative of the highest level of apoptosis for each section. A total of six such images from each animal for each genotype were compared from two independent and replicable experiments.

### Genetic mapping.

Linkage was implicated by the association of phenotype with homozygosity for C57B6/J, the parental strain that was mutagenized. Additionally, heterozygous animals were crossed to wild-type CAST/Ei animals to take advantage of the higher degree of polymorphism between C57BL/6J and CAST/Ei.

### Genotyping.

Approximately 2-mm tail tips were lysed and directly used as template in subsequent standard PCR reactions. PCR products were separated by electrophoresis on a horizontal 4.0% MetaPhor (Cambrex, http://www.cambrex.com) gel.

### RT-PCR, cDNA sequencing, and expression analysis.

C57BL/6J +/+ and *mei4*/*mei4* mutant total RNA was extracted from homogenized testis tissue with RNeasy Mini kit (Qiagen, http://www1.qiagen.com). We then used 3 μg of total RNA as template for RT-PCR that was performed using First-strand cDNA Synthesis kit (Fermentas, http://www.fermentas.com). We amplified 1.5 μl of normalized wild-type and *mei4* mutant cDNA in parallel 25-μl PCR reactions to see whether expression of these genes was different in wild-type and *mei4* mutant animals. Expression analysis was performed using primers specific for *Parp-2, Apex1, Hei10* (Table S2, MEI10cDNA1F.2 and MEI10cDNA2R.1), and *beta*-actin (*Actb*). Amplifications of *Hei10* mutant transcript were then isolated using the QIAquick PCR Purification kit and bidirectionally sequenced on an Applied Biosystems 3130 Automated sequencer (Applied Biosystems, http://www.appliedbiosystems.com). Relative tissue expression was then analyzed by PCR and electrophoresis with a normalized Multiple Tissue cDNA Panel (BD Biosciences). PanelcDNAEx1F.1 and PanelcDNAExon3R primers (Table S2) were designed to match manufacturer's suggested product size and annealing temperature. *Gapdh* controls were amplified with 22 cycles while *Hei10* transcripts were amplified with 37 cycles using manufacturers cycling parameters.

### Genomic sequencing.

Sixteen PARP-2 exons, five APEX1 exons, and three *Hei10* exons were amplified from genomic DNA template, and products were then isolated with the QIAquick Gel Extraction kit or PCR purification kit (Qiagen). Mutant sequences (in the case of *Hei10*) were identified and sequencing analysis was repeated with another mutant individual and one heterozygote and compared to sequence gathered from C57BL/6J, Cast/Ei, and C3HeB/FeJ control mice. MEI10Ex1F and MEI10Ex1R (Table S2) were used to amplify and sequence the first exon of *Hei10*.

### Statistical analysis.

To determine significance in our apoptotic cell counts we used a one-tailed, unequal variance Student's *t*-test. Significance during in vitro fertilization and blastocyst culture was also determined using a one-tailed *t*-test.

## Supporting Information

Table S1 Ensembl (Version 43) Predicted Genes Between D14Mit101 and D14Mit183 (123 KB DOC)

Table S2 Primers (5′–3′) (40 KB DOC)

### Accession Numbers

The Ensembl (http://www.ensembl.org) sequence accession information for HEI10 and its orthologs discussed in this paper are as follows: H. sapiens, ENSP00000337396; M. musculus, ENSMUSP00000093622; *C. familiaris,* ENSCAFP00000008029; B. Taurus, 1ENSBTAP00000012337; M. domestica, ENSMODP00000008960; ENSOANP00000001628; X. tropicalis, ENSXETP00000037001; G. aculeatus, ENSGACP00000025421; D. rerio, XP_691290; C. intestinalis, ENSCINP00000019943; and S. purpuratus, XP_780975. The Ensembl Gene ID for Gm288 is ENSMUSG00000071470.

## Abbreviations

*Apex1*: apurinic/apyrimidic endonuclease 1
CCNB1: cyclin B1
CCNB3: cyclin B3
CDK: cyclin dependent kinase
DAPI: 4′, 6-diamidino-2-phenylindole, dihydrochloride
DSB: double strand break
*Hei10*: Human Enhancer of Invasion 10
MI: meiosis I
MLH: MutL homologs
MMR: mismatch repair
MSH: MutS homolog
RT: reverse transcriptase
SC: synaptonemal complex
