# Supplementary material for: Mutation in Mouse Hei10, an E3 Ubiquitin Ligase, Disrupts Meiotic Crossing Over
Source: PLoS Genet. 2007 Aug 31;3(8):e139. doi: 10.1371/journal.pgen.0030139 (PMC1959360; doi:10.1371/journal.pgen.0030139)
Supplement: Supplementary file 1 [file pgen.0030139.st001.doc]

| **Supplementary Table 1.** Ensembl (v43) Predicted Genes Between D14Mit101 and D14Mit183 | | | | | |
| --- | --- | --- | --- | --- | --- |
|  |  |  |  |  |  |
|  | **Start (bp)** | **End (bp)** | **Ensembl Gene ID** | **External Gene ID** | **RefSeq ID** |
| 1 | 48524380 | 48524547 | ENSMUSG00000068440 | *Q78DY9_MOUSE* |  |
| 2 | 48604299 | 48696196 | ENSMUSG00000036242 | *3632451O06Rik* | NM_026142 |
| 3 | 48816209 | 48823892 | ENSMUSG00000048933 | *Olfr722* | NM_146494 |
| 4 | 48850564 | 48851493 | ENSMUSG00000062622 | *Olfr723* | NM_001011530 |
| 5 | 48882092 | 48883021 | ENSMUSG00000058227 | *Olfr724* | NM_146492 |
| 6 | 48956387 | 48957352 | ENSMUSG00000068437 | *Olfr725* | NM_146317 |
| 7 | 49005665 | 49006630 | ENSMUSG00000060523 | *Olfr726* | NM_146316 |
| 8 | 49048530 | 49049504 | ENSMUSG00000059488 | *Olfr727* | NM_146319 |
| 9 | 49061653 | 49062588 | ENSMUSG00000050030 | *Olfr728* | NM_001011809 |
| 10 | 49069852 | 49070823 | ENSMUSG00000049011 | *Olfr729* | NM_146278 |
| 11 | 49108213 | 49109169 | ENSMUSG00000050431 | *Olfr730* | NM_146493 |
| 12 | 49159854 | 49160834 | ENSMUSG00000048080 | *Olfr731* | NM_146363 |
| 13 | 49203276 | 49204202 | ENSMUSG00000057782 | *Olfr732* | NM_146665 |
| 14 | 49220332 | 49221258 | ENSMUSG00000056813 | *Olfr733* | NM_146663 |
| 15 | 49241843 | 49242784 | ENSMUSG00000045306 | *Olfr734* | NM_146664 |
| 16 | 49267327 | 49268391 | ENSMUSG00000046210 | *Olfr735* | NM_001011754 |
| 17 | 49282453 | 49285445 | ENSMUSG00000051969 | *Tlr11* | NM_205819 |
| 18 | 49314709 | 49315647 | ENSMUSG00000047716 | *Olfr736* | NM_146666 |
| 19 | 49335497 | 49336432 | ENSMUSG00000049601 | *Olfr738* | NM_146420 |
| 20 | 49346472 | 49347401 | ENSMUSG00000057903 | *Olfr739* | NM_146668 |
| 21 | 49375005 | 49375940 | ENSMUSG00000068433 | *Olfr740* | NM_146667 |
| 22 | 49407411 | 49408346 | ENSMUSG00000060108 | *Olfr741* | NM_207133 |
| 23 | 49437157 | 49438092 | ENSMUSG00000068431 | *Olfr742* | NM_146430 |
| 24 | 49455365 | 49456300 | ENSMUSG00000062237 | *Olfr265* |  |
| 25 | 49540175 | 49541146 | ENSMUSG00000053815 | *Olfr744* | NM_001011738 |
| 26 | 49564234 | 49565220 | ENSMUSG00000050028 | *Olfr745* | NM_146299 |
| 27 | 49575190 | 49576134 | ENSMUSG00000058188 | *Olfr746* | NM_146298 |
| 28 | 49602642 | 49603583 | ENSMUSG00000057179 | *Olfr747* | NM_207156 |
| 29 | 49632283 | 49633227 | ENSMUSG00000060084 | *Olfr748* | NM_001011837 |
| 30 | 49653004 | 49653110 | ENSMUSG00000064975 |  |  |
| 31 | 49658170 | 49659141 | ENSMUSG00000059069 | *Olfr749* | NM_020288 |
| 32 | 49687362 | 49707461 | ENSMUSG00000006288 | *Ttc5* | NM_177625 |
| **33** | **49711764** | **49715805** | **ENSMUSG00000071470** | ***Gm288*** |  |
| 34 | 49729435 | 49729543 | ENSMUSG00000076225 | *mmu-mir-685* |  |
| **35** | **49729897** | **49743251** | **ENSMUSG00000036023** | ***Parp2*** | **NM_009632** |
| 36 | 49746013 | 49792511 | ENSMUSG00000006281 | *Tep1* | NM_009351 |
| 37 | 49777557 | 49875363 | ENSMUSG00000021871 |  |  |
| 38 | 49777557 | 49875363 | ENSMUSG00000021871 | *Pnp* | NM_013632 |
| 39 | 49813460 | 49818873 | ENSMUSG00000072578 |  |  |
| 40 | 49828429 | 49846785 | ENSMUSG00000006289 | *Osgep* | NM_133676 |
| **41** | **49846929** | **49849089** | **ENSMUSG00000035960** | ***Apex1*** | **NM_009687** |
| 42 | 49848021 | 49852800 | ENSMUSG00000035953 | *Tmem55b* | NM_001033271 |
| 43 | 49878088 | 49886470 | ENSMUSG00000068417 | *Pnp* |  |
| 44 | 49900395 | 49900887 | ENSMUSG00000052408 |  |  |
| 45 | 49929702 | 49932712 | ENSMUSG00000021872 | *Rnase10* | NM_029145 |
| 46 | 49960410 | 49963820 | ENSMUSG00000052382 | *Rnase9* | NM_183032 |
| 47 | 49971468 | 49972046 | ENSMUSG00000059648 | *Rnase11* | NM_001011877 |
| 48 | 49978734 | 49979171 | ENSMUSG00000068407 | *Rnase12* | NM_001011875 |
| 49 | 49992347 | 49993342 | ENSMUSG00000035932 | *Olfr750* | NM_207558 |
| 50 | 50013068 | 50028097 | ENSMUSG00000021876 | *Rnase4* | NM_021472 |
| 51 | 50018364 | 50023962 | ENSMUSG00000072115 | *Ang1* | NM_007447 |
| 52 | 50038508 | 50038957 | ENSMUSG00000072575 | *Gm75* | NM_203508 |
| 53 | 50051041 | 50052975 | ENSMUSG00000021880 | *Rnase6* | NM_030098 |
| 54 | 50066953 | 50068718 | ENSMUSG00000035896 | *Rnase1* | NM_011271 |
| 55 | 50084415 | 50084882 | ENSMUSG00000059606 | *Ear5* | NM_019398 |
| 56 | 50117437 | 50117874 | ENSMUSG00000047894 | *Ang2* | NM_007449 |
| 57 | 50125640 | 50126107 | ENSMUSG00000050766 | *Ear14* | NM_001017422 |
| 58 | 50177212 | 50178063 | ENSMUSG00000047222 | *Ear11* | NM_053113 |
| 59 | 50283707 | 50446121 | ENSMUSG00000068399 | *NP_001034339.1* | NM_001039250 |
| 60 | 50333025 | 50341478 | ENSMUSG00000000606 | *V2r3* | XR_002406 |
| 61 | 50373993 | 50379536 | ENSMUSG00000070448 | *V2r11* |  |
| 62 | 50576738 | 50579837 | ENSMUSG00000072574 | *NP_001013838.1* | NM_001013816 |
| 63 | 50633595 | 50639083 | ENSMUSG00000068506 | *NP_001029274.1* | NM_001034102 |
| 64 | 50685838 | 50695541 | ENSMUSG00000060615 | *Ang4* | NM_177544 |
| 65 | 50721997 | 50748247 | ENSMUSG00000050961 | *NP_919328.1* | NM_194347 |
| 66 | 50775719 | 50776594 | ENSMUSG00000062148 | *Ear6* | NM_053111 |
| 67 | 50806886 | 50814990 | ENSMUSG00000004561 | *D14Ertd209e* | NM_001029990 |
| 68 | 50815610 | 50818696 | ENSMUSG00000072572 | *Slc39a2* | NM_001039676 |
| 69 | 50827232 | 50835302 | ENSMUSG00000004558 | *Ndrg2* | NM_013864 |
| 70 | 50840376 | 50842650 | ENSMUSG00000008813 | *XP_992198.1* |  |
| 71 | 50844170 | 50844631 | ENSMUSG00000068392 | *Rnase13* | NM_001011687 |
| 72 | 50909354 | 50927579 | ENSMUSG00000004562 | *E130112L23Rik* | NM_198249 |
| 73 | 50928037 | 50931616 | ENSMUSG00000049295 | *Zfp219* | NM_027248 |
