# Supplementary material for: Mutation in Mouse Hei10, an E3 Ubiquitin Ligase, Disrupts Meiotic Crossing Over
Source: PLoS Genet. 2007 Aug 31;3(8):e139. doi: 10.1371/journal.pgen.0030139 (PMC1959360; doi:10.1371/journal.pgen.0030139)
Supplement: Supplementary file 2 [file pgen.0030139.st002.doc]

| **Supplementary Table 2.** Primers (5’-3’) | | | |  |
| --- | --- | --- | --- | --- |
| ***Hei10* Sequencing Primers** | | | |  |
| MEI10Ex1F |  | | TTTGGAGATTCGCCATGTCTTTGTG |  |
| MEI10Ex1R |  | | GTGGAGGGTACTGTGGCCTATCAGG |  |
| MEI10Ex2F |  | | GCCGGTTTTCTGTAGTACGCTCATTG |  |
| MEI10Ex2R |  | | TTCGCCCTCAAAGACTTCTGAATG |  |
| MEI10Ex3F |  | | CATGGTAGGGGAAAGCGGAGTGAC |  |
| MEI10Ex3R |  | | CACACGCCCTACTGCAAGCCAAC |  |
| ***Hei10* Expression and 5’ Transcript Primers** | | | |  |
| MEI10cDNA1F.2 | |  | CCTGCTTGCAACAGTACCCTTTCTGG |  |
| MEI10cDNA2R.1 | |  | GTGGGAAGCCAAAGACTCCAGACTG |  |
| PanelcDNAEx1F.1 | |  | GACATTAGCTCCCGGGCATTGGCCTTCTG |  |
| PanelcDNAExon3R | |  | TGCTGCTCTGCTTCATGGCTTGGAGATGC |  |
